# NPD-0414-2 and NPD-0414-24, Two Chemical Entities Designed as Aryl Hydrocarbon Receptor (AhR) Ligands, Inhibit Gut Inflammatory Signals

**DOI:** 10.3389/fphar.2019.00380

**Published:** 2019-04-12

**Authors:** Irene Marafini, Davide Di Fusco, Vincenzo Dinallo, Eleonora Franzè, Carmine Stolfi, Giuseppe Sica, Giovanni Monteleone, Ivan Monteleone

**Affiliations:** ^1^ Department of Systems Medicine, Gastroenterology, University of Tor Vergata, Rome, Italy; ^2^ Department of Surgery, University of Tor Vergata, Rome, Italy; ^3^ Department of Biomedicine and Prevention, University of Tor Vergata, Rome, Italy

**Keywords:** AhR, Ficz, IBD, Crohn’s disease, ulcerative colitis, Il-22, IFN-γ, TNBS

## Abstract

Defects in counterregulatory mechanisms contribute to amplify the detrimental inflammatory response leading to the pathologic process occurring in the gut of patients with Crohn’s disease (CD) and ulcerative colitis (UC), the major inflammatory bowel diseases (IBDs), in human beings. One such mechanism involves aryl hydrocarbon receptor (AhR), a transcription factor activated by natural and synthetic ligands, which induces the production of interleukin (IL)-22 and down-regulates inflammatory signals. In IBD, AhR expression is down-regulated and its activation by natural ligands promotes clinical and endoscopic benefit. Since the use of AhR natural ligands can associate with serious adverse events, we developed new chemical ligands of AhR and assessed their regulatory effects. Among these derivatives, we selected the compounds NPD-0414-2 and NPD-0414-24, as they displayed the more pronounced capacity to induce IL-22. Peripheral blood mononuclear cells and lamina propria mononuclear cells (LPMC) were isolated from CD and UC patients. Cells were treated *in vitro* with Ficz, AhR ligands, and AhR antagonist and then cytokines’ expression was evaluated by real-time PCR and flow cytometry. After the induction of TNBS colitis, Ficz and AhR ligands were injected intra-peritoneally to wild type and AhR knock-out mice. After 4 days, mice were sacrificed and colonic tissues were collected for histologic examination and real-time PCR analysis. Treatment of IBD LPMC with NPD-0414-2 and NPD-0414-24 reduced IFN-γ and increased IL-22 transcripts, and these effects were abrogated by CH223191, a specific inhibitor of AhR interaction with its ligands. Mice given NPD-0414-2 and NPD-0414-24 developed a significantly less severe form of TNBS colitis and exhibited reduced expression of IFN-γ and increased expression of IL-22. The therapeutic effect of NPD-0414-2 and NPD-0414-24 on the ongoing colitis was abrogated in AhR-deficient mice. Collectively, these data show that NPD-0414-2 and NPD-0414-24 exert Ahr-dependent regulatory effects in the gut.

## Introduction

Crohn’s disease (CD) and ulcerative colitis (UC) are chronic inflammatory diseases of the gastrointestinal tract characterized by various degrees of tissue damage (Torres et al., 2017; Ungaro et al., 2017). Although the cause of CD and UC remains unknown, accumulating evidence suggests that both inflammatory bowel diseases (IBDs) arise in genetically predisposed individuals as a result of the action of many environmental insults, which trigger an abnormal mucosal immune inflammatory response that is poorly controlled by counterregulatory mechanisms (Kaser et al., 2010; Neurath, 2014; De Souza and Fiocchi, 2016). Consistently, drugs that either block effector cell pathways or enhance anti-inflammatory signals have largely contributed to combat the IBD-associated pathological process (Danese et al., 2015). Unfortunately, not all IBD patients respond to conventional, immune suppressors, and biologic drugs and the use of these drugs can enhance the risk of adverse events, thus, suggesting the necessity of further treatments. In this context, studies in pre-clinical models of colitis can help identify novel compounds, which have the ability to suppress detrimental signals in a safe manner.

Aryl hydrocarbon receptor (AhR), a member of the Pern-Arnt-Sim (PAS) superfamily of transcription factors activated by a large variety of natural and synthetic ligands (Kewley et al., 2004), is considered as a promising therapeutic target in immune-mediated diseases (Gutierrez-Vazquez and Quintana, 2018). In the gut, AhR controls the differentiation, activation, and proliferation of many immune cells and triggers anti-inflammatory pathways such as the development of T-regulatory (T-reg) cells or interleukin (IL)-10 expressing T-reg type 1 cells (Apetoh et al., 2010; Gutierrez-Vazquez and Quintana, 2018). We have previously shown that activation of AhR in intestinal mucosal cells with the AhR agonist 6-formylindolo(3, 2-b)carbazole (Ficz) induces the production of IL-22 and down-regulates the expression of inflammatory cytokines (e.g. IFN-γ), with the down-stream effect of attenuating experimental colitis in mice (Monteleone et al., 2011). Moreover, AhR has been recently identified as a candidate gene for associated IBD susceptibility loci (Liu et al., 2015). In a randomized, placebo-controlled trial, Naganuma and colleagues showed that 8-week treatment with indigo naturalis, a traditional Chinese medicine that contains ligands for AhR, was effective in inducing clinical and endoscopic benefit in patients with UC (Sugimoto et al., 2016; Naganuma et al., 2018). However, the wide and long-term use of this compound could be hampered by the risk of emerging adverse effects, such as pulmonary arterial hypertension and liver dysfunction, even though it remains unclear if the side effects documented during indigo naturalis treatment are either strictly dependent on AhR activation or reflecting the modulation of other intracellular pathways. Therefore, the validation of novel AhR activators coupling good safety profiles and enhanced anti-inflammatory activity would be highly desirable. To this end, we have developed several structurally related Ficz compounds, and initially assessed their effect on the induction of IL-22 by blood mononuclear cells. Among these derivatives, we selected the compounds NPD-0414-2 and NPD-0414-24, as they displayed the more pronounced capacity to induce IL-22. Next, we tested the modulatory effects of such compounds in human gut immune cells and in an experimental mouse model of colitis.

## Materials and Methods

### Patients and Samples

Mucosal biopsies were obtained from involved colonic or ileum areas of nine patients with active CD undergoing endoscopy (median age, 38 years; range, 25–61 years). Mucosal biopsies were also obtained from involved areas of seven patients with active UC undergoing endoscopy (median age, 35 years; range, 23–56 years). Intestinal specimens were taken from patients with moderate to severe CD undergoing intestinal resection and from UC patients undergoing colectomy for a severe disease poorly responsive to medical treatment. Six patients with CD were receiving corticosteroids, while three patients were treated with mesalazine. Four patients with UC were receiving corticosteroids, while three patients were treated with mesalazine. Each patient who took part in the study gave written informed consent and the independent local Ethics Committee of the University hospital of Tor Vergata approved the study protocol.

### New Chemical Ligands

An *in silico* scaffold hopping approach was used to discover new chemical entity, starting from the lowest energy conformation of β-carboline derivatives (shown *in vitro* to increase AhR signaling), and replacing the central β-carboline core. Upon *in silico* ADMET screening, the new chemical ligands were synthetized for *in vitro* screening.

### Cell Isolation and Culture

Peripheral blood mononuclear cells (PBMCs) were isolated from EDTA-stabilized peripheral blood samples of CD patients and UC patients by Ficoll gradients, pre-incubated for 1 h with Ficz (final concentration 200 nmol; Alexis), NDP-0614-2, NDP-0614-4, NDP-0614-13, NDP-0614-15, NDP-0614-17, and NDP-0614-24 (final concentration 200 nM), then stimulated with activating anti-CD3/CD28 beads (Miltenyi Biotec, Calderara di Reno, Italy) for 18 and analyzed by RT-PCR.

Human LPMC were isolated as previously described with minor modifications (Monteleone et al., 2010). Briefly, samples taken from CD patients and UC patients were freed of mucus with dithiothreitol (DTT). Cells were treated with ethylenediaminetetraacetic acid (EDTA) to separate epithelial cells from the lamina propria. The remaining tissue was digested with liberase™ (0.2 mg/ml; Roche, Mannheim, Germany) and DNase I (0.2 mg/ml; Roche). LPMC were resuspended (1 × 10^6^/ml) in RPMI-1640 supplemented with 10% fetal bovine serum, penicillin (100 μg/ml), streptomycin (100 μg/ml), and gentamycin (50 μg/ml; Lonza, Milan, Italy). Cells were pre-incubated with Ficz (final concentration 200 nmol/l; Alexis, Milan, Italy), NDP-0614-2 and NDP-0614-24 (final concentration 50, 100, and 200 nM), then stimulated with activating anti-CD3/CD28 beads (Miltenyi Biotec, Calderara di Reno, Italy) for 18 and 36 h and analyzed by RT-PCR and flow cytometry. Cells were also pre-incubated with 2-methyl-2H-pyrazole-3-carboxylic acid (CH223191), an AhR antagonist (final concentration 10 μM; Calbiochem, Nottingham, England) for 1 h, stimulated with Ficz (final concentration 200 nmol), NDP-0614-2 (final concentration 200 nmol), NDP-0614-24 NDP-0614-13 (final concentration 200 nmol), and then with activating anti-CD3/CD28 beads for 18 h and analyzed by RT-PCR. In each experiment, cell viability was evaluated using flow-cytometry. Phorbol myristate acetate (PMA, 10 ng/ml), ionomycin (1 mg/ml), and brefeldin A (10 mg/ml, eBioscience, San Diego, CA) were added to the cultures in the last 3 h in order to evaluate cytokines’ production.

### RNA Extraction, Complementary DNA Preparation, and Real-Time Polymerase Chain Reaction

RNA isolation, reverse transcription of the RNA, and real-time PCR were carried out as previously described. RNA was extracted by using TRIzol reagent according to the manufacturer’s instructions (Invitrogen, Carlsbad, CA, USA). A constant amount of RNA (1 μg/sample) was reverse transcribed into complementary DNA, and this was amplified using the following conditions: denaturation for 1 min at 95°C; annealing for 30 s at 58°C for human IFN-γ, mouse TNF-α, mouse β-defensin, 57°C for mouse MUC1, and 59°C for mouse MUC3, at 60°C for human and mouse β-actin and mouse IFN-γ, followed by 30 s of extension at 72°C. IL-22 was evaluated using commercially available TaqMan probes (Applied Biosystems, Foster City, CA). All real-time PCR data were normalized to β-actin. Gene expression was calculated using the ΔΔCt algorithm.

### Flow Cytometry

Cells were immunostained with the following monoclonal anti-human antibodies: APC-H7 anti-CD45, APC anti-IFN-γ, and PE anti-IL-22 (BD Bioscience, San Jose, CA). In all experiments, appropriate isotype control IgGs (BD Bioscience) and fluorescence minus one controls were used. All antibodies were used at 1:100 final dilution. For intracellular immunostaining, cells were fixed and permeabilized using staining buffer set and permeabilization buffer (both from eBioscience) according to the manufacturer’s instruction. Cells were analyzed by flow cytometry (FACSverse, BD Bioscience). To assess cell viability, LPMC were cultured as indicated above, and the percentage of propidium iodide(PI)-positive cells was assessed by flow cytometry according to the manufacturer’s instructions (Immunotech, Marseille, France).

### TNBS Colitis

About 2 mg of TNBS in 35% ethanol was administered to 8-week-old female wild type (WT) and AhR knock-out (KO) mice on a C57BL/6 back-ground as previously described (Wirtz et al., 2007). Controls consisted of mice treated with 35% ethanol. Ficz (2.5 μg/mouse), NDP-0614-2 (1.25, 2.5, or 5 μg/mouse) and NDP-0614-24 (1.25, 2.5 or 5 μg/mouse) were injected intra-peritoneally on the 2nd and 3rd day after TNBS administration to WT and AhR KO mice. Ficz and AhR ligands were dissolved in 10 μl dimethyl sulfoxide, and this solution was then mixed with 140 μl phosphate-buffered saline. Weight changes were recorded daily, mice were killed at day 4, and tissues were collected for histology and RNA analysis. All animal experiments were approved by the local animal ethics committee (n° 647/2016-PR) according to Italian legislation on animal experiments.

### Statistical Analysis

Differences between groups were compared using Student’s *t* test. All analyses were performed using GraphPad Prism version 5.00 software for Windows (GraphPad Software, San Diego California, USA, www.graphpad.com).

## Results

### NPD-0414-2 and NPD-0414-24 Increase IL-22 and Reduce IFN-γ Expression in IBD LPMC

**Figure 1:**
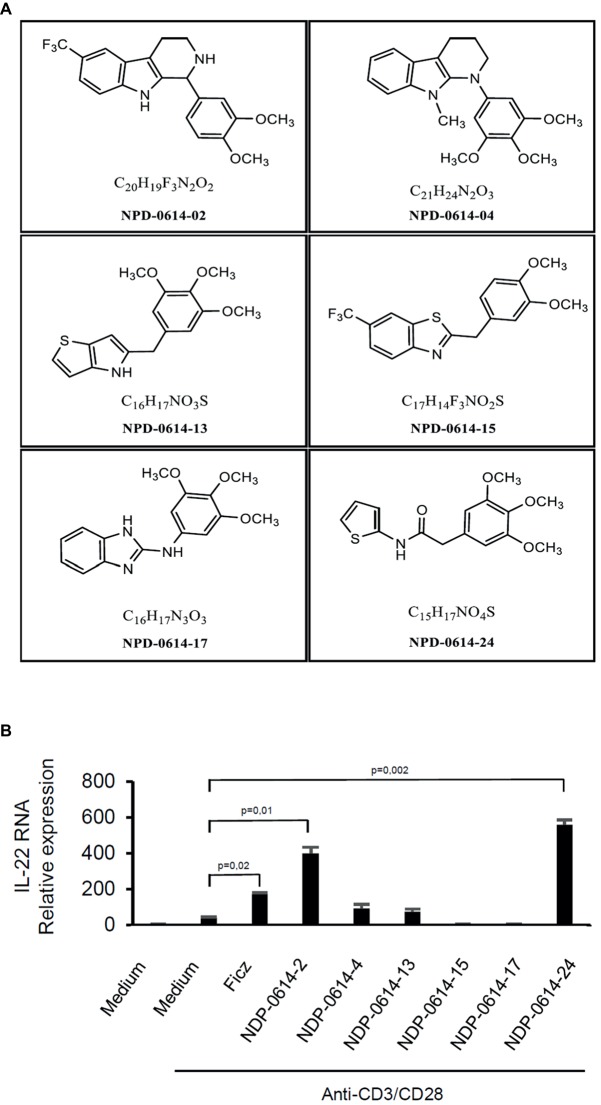
**(A)** Schematic representation of the chemical structure of new chemical ligands of AhR. **(B)** NPD-0414-2 and NPD-0414-24 induce IL-22 expression in human peripheral blood mononuclear cells (PBMC) of inflammatory bowel disease (IBD) patients. PBMC were isolated from the peripheral blood of IBD patients and stimulated *in vitro* with anti-CD3/CD28 beads, Ficz and the six chemical ligands of AhR. IL-22 RNA expression was evaluated by real time-PCR and normalized to β-actin. Histograms are representative of five separate experiments; values are expressed as mean ± SEM.

**Figure 2:**
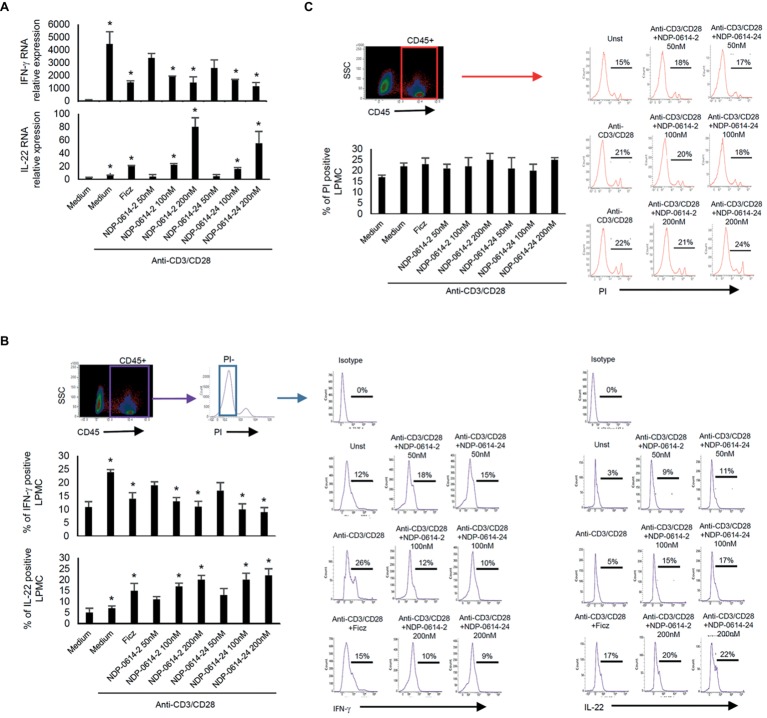
NPD-0414-2 and NPD-0414-24 reduce IFN-γ and increase IL-22 expression in lamina propria mononuclear cells (LPMC) of inflammatory bowel disease (IBD) patients. **(A)** IBD LPMC were stimulated *in vitro* with anti-CD3/CD28 beads and 1 h later were treated with graded doses of NPD-0414-2, NPD-0414-24 or Ficz. After 18 h, IFN-γ and IL-22 RNA transcripts were analyzed by real time-PCR. Histograms are representative of seven separate experiments; values are expressed as mean ± SEM (*anti-CD3/CD28 vs all AhR ligands *p* ≤ 0.04). **(B)** IBD LPMC were stimulated *in vitro* as above and IFN-γ and IL-22 expressing cells were analyzed after 36 h by flow-cytometry. Histograms are representative of seven separate experiments; values are expressed as mean ± SEM (*anti-CD3/CD28 vs all AhR ligands *p* ≤ 0.05). Representative flow-cytometry plots are also shown. CD45 positive, propidium iodide (PI)-negative cells were analyzed for cytokines’ expression; numbers indicate the percentage of IFN-γ and IL-22 positive cells. **(C)** Histograms showing CD45 positive PI-positive IBD LPMC stimulated as indicated in A for 36 h. Histograms are representative of seven separate experiments; values are expressed as mean ± SEM. Representative flow-cytometry plots are also shown; numbers indicate the percentage of PI cells.

In initial experiments, we tested the effect of several Ficz-related derivatives on the induction of IL-22 in activated IBD PBMC (Figure 1), as we previously showed that the anti-inflammatory properties of AhR rely mainly on the induction of such a cytokine (Monteleone et al., 2011). Among these compounds, NPD-0414-2 and NPD-0414-24 displayed the more pronounced capacity to induce IL-22 RNA expression; interestingly, IL-22 RNA expression was at least twice greater in cells treated with either NPD-0414-2 or NPD-0414-24 than in those treated with Ficz (Figure 1B). Therefore, we selected these two compounds for the subsequent experiments with IBD mucosal cells. Treatment of anti-CD3 + CD28 IBD LPMC with each of these two derivatives reduced IFN-γ RNA expression and increased IL-22 RNA expression in a dose-dependent fashion (Figure 2A). Consistently, flow cytometry analysis showed that NPD-0414-2 and NPD-0414-24 significantly reduced the percentages of IFN-γ-expressing IBD LPMC and increased the fractions of IL-22-producing IBD LPMC (Figure 2B). No significant change in cell death was seen after the treatment of IBD LPMC with either NPD-0414-2 or NPD-0414-24 (Figure 2C).

### CH223191, a Specific Inhibitor of the Interaction Between AhR and its Ligands, Reverts the Anti-Inflammatory Effects of NPD-0414-2 and NPD-0414-24

**Figure 3:**
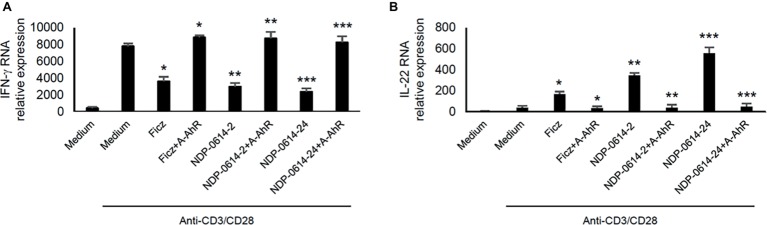
AhR antagonist abrogates the effect of NPD-0414-2 and NPD-0414-24 on IFN-γ and IL-22 expression in lamina propria mononuclear cells (LPMC) of inflammatory bowel disease (IBD) patients. **(A**,**B)** IBD LPMC were stimulated *in vitro* with anti-CD3/CD28 beads and 1 hour later were treated with NPD-0414-2, NPD-0414-24 (final concentration 200 nmol) or Ficz with or without the AhR antagonist (A-AhR, final concentration 10 μmol). After 18 h, IFN-γ and IL-22 RNA transcripts were analyzed by real time-PCR. Histograms are representative of 5 separate experiments; values are expressed as mean ± SEM (*Fics vs AhR antagonist *p* < 0.05, ** NPD-0414-2 vs AhR antagonist *p* < 0.03, *** NPD-0414-24 vs AhR antagonist *p* < 0.01).

To confirm that the regulatory effect of NPD-0414-2 and NPD-0414-24 on cytokine expression was strictly dependent on the activation of AhR, anti-CD3/CD28-activated IBD LPMC were treated with Ficz, NPD-0414-2 or NPD-0414-24 in the presence or absence of CH223191, a specific inhibitor of the interaction between AhR and its ligands. Pre-incubation of IBD LPMC with CH223191 fully abolished the regulatory effect of Ficz, NPD-0414-2, and NPD-0414-24 on IFN-γ and IL-22 expression (Figure 3).

### NPD-0414-2 and NPD-0414-24 Ameliorate TNBS-Induced Colitis

**Figure 4:**
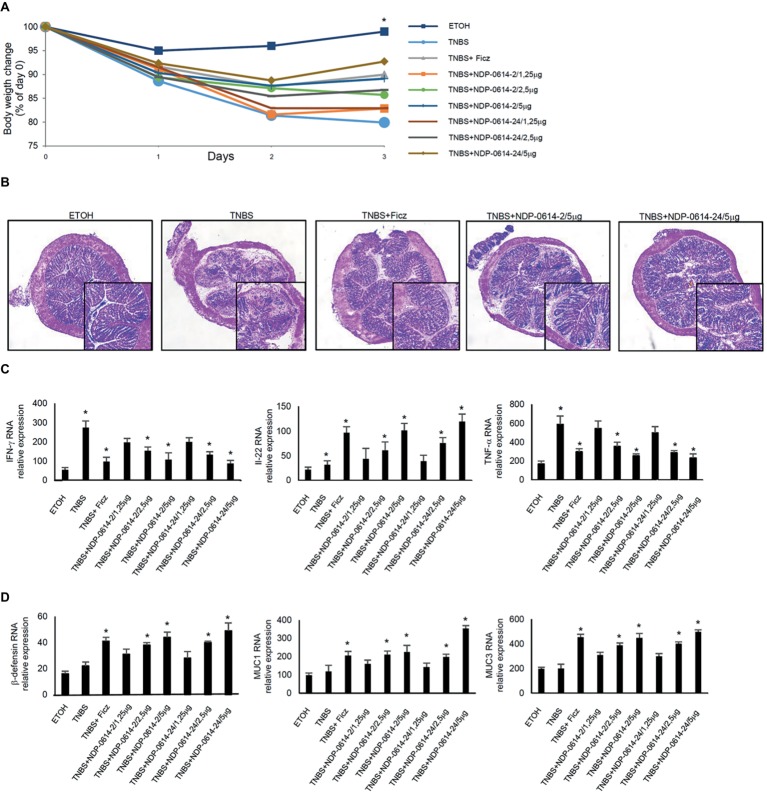
NPD-0414-2 and NPD-0414-24 ameliorate TNBS colitis. **(A)** Mice were rectally administered with TNBS and, at days 1 and 2, were intraperitoneally given graded doses of NPD-0414-2 or NPD-0414-24 or Ficz. Control mice were treated with ethanol (ETOH) alone. Mice were sacrificed at day 4. Body weight was recorded daily and each point on the graph indicates cumulative mean of three separate experiments (*TNBS vs all AhR ligands at final concentration 2.5 or 5 μg/mouse *p* < 0.05). **(B)** Representative H&E-stained colonic sections of mice receiving ETOH alone, TNBS+PBS, TNBS+Ficz, TNBS+NPD-0414-2 or TNBS+NPD-0414-24. The insets represent a higher magnification. **(C)** Colonic tissues were collected from mice of the different groups and IFN-γ, IL-22 and TNF-α RNA transcripts were analyzed by real-time PCR. Data are shown as mean ± SD of three separate experiments (*n* = 12 mice total per group). (*TNBS vs all AhR ligands at final concentration 2.5 or 5 μg/mouse *p* < 0.04). **(D)** Colonic tissues were collected from mice of the different groups and β-defensin, MUC1 and MUC3 RNA transcripts were analyzed by real-time PCR. Data are shown as mean ± SD of three separate experiments (*n* = 12 mice total per group). (*TNBS vs all AhR ligands at final concentration 2.5 or 5 μg/mouse *p* ≤ 0.04).

To translate these data *in vivo*, we used an experimental mouse model of colitis, where mice were given Ficz or graded doses of NPD-0414-2 and NPD-0414-24 1 day after TNBS administration. Mice given NPD-0414-2 and NPD-0414-24 exhibited no clinical sign of systemic toxicity. TNBS-treated mice exhibited a significant weight loss as compared to ethanol-treated mice (controls) (Figure 4A). In line with our previous data, at day 4, TNBS-treated mice given Ficz had significant less body weight loss (Figure 4A). Similarly, both NPD-0414-2 and NPD-0414-24 dose-dependently reduced body weight loss when administered to colitic mice (Figure 4A). Consistently, histologic examination of colonic tissues revealed that activation of AhR with either Ficz or each of the two derivatives led to attenuation of colitis (Figure 4B), which was associated with a significant reduction of IFN-γ and TNF-α and up-regulation of IL-22 RNA expression (Figure 4C). Moreover, to better characterize the effect of Ahr ligand administration on the IL-22 downstream pathways, we analyzed by RT-PCR β-defensin, MUC1, and MUC3 in the colon mucosa. As indicated in Figure 4D, administration of AhR ligands was associated with a significant increase of anti-microbial factors such as β-defensin, MUC1, and MUC3 (Figure 4D).

### AhR-KO Mice are Resistant to the Therapeutic Effect of NPD-0414-2 and NPD-0414-24

**Figure 5:**
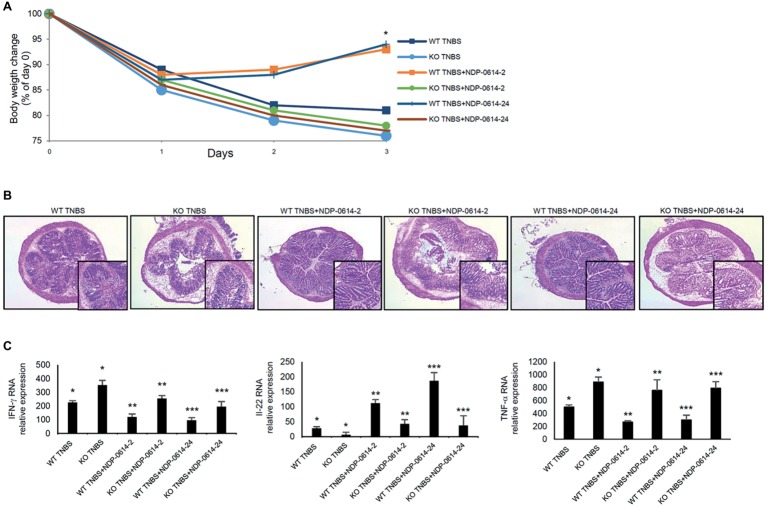
The therapeutic effect of NPD-0414-2 and NPD-0414-24 on the TNBS-colitis is abrogated in AhR knock-out (KO) mice. **(A)** Wild type (WT) and AhR KO mice were rectally administered with TNBS and, at days 1 and 2, were intraperitoneally given graded doses of NPD-0414-2 or NPD-0414-24 or Ficz. Body weight was recorded daily and each point on the graph indicates cumulative mean of three separate experiments (*WT TNBS+NPD-0414-2 vs KO TNBS+NPD-0414-2 and WT TNBS+NPD-0414-24 vs KO TNBS+NPD-0414-24 *p* < 0.05). **(B)** Representative H&E-stained colonic sections of WT and KO mice receiving TNBS+PBS, TNBS+NPD-0414-2 or TNBS+NPD-0414-24. The insets represent a higher magnification. **(C)** Colonic tissues were collected from mice of the different groups and IFN-γ, IL-22, and TNF-α RNA transcripts were analyzed by real-time PCR. Data are shown as mean ± SD of three separate experiments (*n* = 12 mice total per group) (*WT TNBS+Ficz vs KO TNBS+Ficz *p* < 0.05** WT TNBS+NPD-0414-2 vs KO TNBS+NPD-0414-2 *p* < 0.04 WT; ***WT TNBS+NPD-0414-24 vs KO TNBS+NPD-0414-24 *p* < 0.04). Values are expressed in arbitrary units (a.u.).

To further prove that the anti-inflammatory effect of NPD-01414-2 and NPD-0414-24 was due to the specific binding with AhR, TNBS-colitis was induced in WT mice and AhR KO mice and then animals were treated with NPD-01414-2 or NPD-0414-24. The therapeutic effect of NPD-01414-2 and NPD-0414-24 was evident in WT mice but not in AhR KO mice (Figures 5A,B). In line with this, no significant change in IFN-γ, TNF-α, and IL-22 RNA expression was seen in AhR-null mice following treatment with each of the two derivatives, thus confirming that AhR is needed for the regulatory effect of both NPD-01414-2 and NPD-0414-24 on the ongoing mucosal immune response (Figure 5C).

## Discussion

In this study, we designed and tested the effect of several structurally related Ficz compounds on the production of effector cytokines by immune cells and experimental colitis. Screening of the derivatives was based upon their ability to induce IL-22 as our previous study showed that the anti-inflammatory effect of AhR in the gut mainly mediated this cytokine, and there is evidence that IL-22 plays a crucial role in maintaining mucosal homeostasis. Among these derivatives, we selected the compounds NPD-0414-2 and NPD-0414-24, as both were more powerful than Ficz in inducing IL-22 in IBD PBMC and LPMC. Additionally, NPD-0414-2 and NPD-0414-24 reduced RNA transcripts for IFN-γ and the percentages of activated IBD LPMC producing such a cytokine. It is unlikely that inhibition of IFN-γ is secondary to IL-22 induction, as IL-22 exerts regulatory effects by targeting non-immune cells (i.e. epithelial cells). Moreover, NPD-0414-2 and NPD-0414-24 did not alter the percentages of propidium iodide-positive LPMC, thus excluding the possibility that the negative effect of these derivatives on IFN-γ relies on induction of cell death. The small number of patients included in the present study did not allow us to ascertain whether concomitant corticosteroid treatment influenced the results. However, we would like to point out that our previous study showed that, in IBD, current therapy did not influence AhR expression (Monteleone et al., 2011).

Next, we confirmed the anti-inflammatory effect of the two derivatives in a well-established model of colitis, namely TNBS-induced colitis. No clinical sign of toxicity was evident in colitic mice given each of these compounds. Both derivatives attenuated the ongoing colitis as evidenced by a reduction in weight loss and of microscopic evidence of inflammation, thus reinforcing the concept that AhR ligands can be useful for treating the IBD-associated detrimental immune response. Although, we performed no *in vitro* study to assess protein-ligand interactions, some observations made in this study indicate that the biological effect NPD-0414-2 and NPD-0414-24 is mediated by AhR. Pre-incubation of IBD LPMC with CH223191, a compound inhibiting interaction of Ahr with its ligands, completely abrogated the effect of NPD-0414-2 or NPD-0414-24 on IL-22 and IFN-γ expression. More interestingly, no therapeutic effect was seen when both derivatives were administered to AhR-deficient mice.

AhR is expressed virtually in all the immune and non-immune cells. The main cellular target of NPD-0414-2 and NPD-0414-24 remains to be ascertained. Similarly, it is unknown whether the anti-inflammatory effect of these derivatives relies either on the induction of IL-22 and down-regulation of T cell-derived cytokines (e.g. IFN-γ and TNF-α), as previously shown for Ficz (Monteleone et al., 2012), or on the control of other mucosal cell types (e.g., epithelial cells, dendritic cells, fibroblasts), which are involved during the effector and healing phases of TNBS-colitis. Despite the main cellular target of NPD-0414-2 and NPD-0414-24 remains unknown, it is likely that these compounds exert an effect on intestinal epithelial barrier. In this context, it is noteworthy to mention that AhR activation proved to have a role on epithelial barrier function in an experimental model of ischemia/reperfusion injury (Liu et al., 2018). Moreover, in mice, AhR is able to influence the intestinal microbial community, which in turn is known to contribute to epithelial integrity (Murray et al., 2016).

Despite the promising success of AhR ligands in different areas of medical research, AhR has been previously considered a liability target for potential treatments of human diseases because of the toxic effects seen following chronic activation of AhR by dioxin exposure (Bradshaw and Bell, 2009). In this context, however, it is noteworthy that several marketed drugs (e.g., leflunomide, prednisolone, omeprazole, and others) can activate AhR, without triggering dioxin-related adverse events; thus, suggesting that binding affinity of various ligands and duration of interaction with AhR can variably influence the regulatory effects and occurrence of adverse events following AhR activation. The present study was not designed to assess the toxicity of NPD-0414-2 and NPD-0414-24, even though in the short time frame of the experimentation mice given these derivatives developed no sign of systemic toxicity. Future studies are needed to evaluate the pharmacokinetic properties of these compounds as well as the potential effects of NPD-0414-2 or NPD-0414-24 on vital functions of the host.

In conclusion, data of the present work confirm and expand on previous results of studies showing the benefit of AhR activation on the course of experimental colitis, supporting the notion that AhR can be a valid target for therapeutic interventions in IBD.

## Ethics Statement

Independent local Ethics Committee of the University hospital of Tor Vergata approved the study protocol.

## Author Contributions

IMo conceived and designed the experiments, analysis and interpretation of data and wrote the paper. IMa, DD, VD, EF, CS, and IMo performed the experiments. GM analysis and interpretation of data and drafting critical revision of the manuscript. GS contributed materials.

### Conflict of Interest Statement

The authors declare that the research was conducted in the absence of any commercial or financial relationships that could be construed as a potential conflict of interest.
